# Combination therapy of insulin‐like growth factor I and BTP‐2 markedly improves lipopolysaccharide‐induced liver injury in mice

**DOI:** 10.1096/fj.202200227RR

**Published:** 2022-07-15

**Authors:** Antoine Nehme, Mahdis Ghahramanpouri, Iqbal Ahmed, Mohadese Golsorkhi, Natasha Thomas, Kevin Munoz, Amir Abdipour, Xiaolei Tang, Sean M. Wilson, Samiksha Wasnik, David J. Baylink

**Affiliations:** ^1^ Division of Regenerative Medicine, Department of Medicine Loma Linda University Loma Linda California USA; ^2^ Pathology and Laboratory Medicine Loma Linda University Loma Linda California USA; ^3^ La Sierra University Riverside California USA; ^4^ Division of Nephrology Loma Linda University Medical Center Loma Linda California USA; ^5^ Department of Veterinary Biomedical Sciences, College of Veterinary Medicine Long Island University Brookville New York USA; ^6^ The Lawrence D. Longo, MD Center for Perinatal Biology, Department of Basic Sciences Loma Linda University School of Medicine Loma Linda California USA

## Abstract

Acute liver injury is a common disease without effective therapy in humans. We sought to evaluate a combination therapy of insulin‐like growth factor 1 (IGF‐I) and BTP‐2 in a mouse liver injury model induced by lipopolysaccharide (LPS). We chose this model because LPS is known to increase the expression of the transcription factors related to systemic inflammation (i.e., NFκB, CREB, AP1, IRF 3, and NFAT), which depends on calcium signaling. Notably, these transcription factors all have pleiotropic effects and account for the other observed changes in tissue damage parameters. Additionally, LPS is also known to increase the genes associated with a tissue injury (e.g., NGAL, SOD, caspase 3, and type 1 collagen) and systemic expression of pro‐inflammatory cytokines. Finally, LPS compromises vascular integrity. Accordingly, IGF‐I was selected because its serum levels were shown to decrease during systemic inflammation. BTP‐2 was chosen because it was known to decrease cytosolic calcium, which is increased by LPS. This current study showed that IGF‐I, BTP‐2, or a combination therapy significantly altered and normalized all of the aforementioned LPS‐induced gene changes. Additionally, our therapies reduced the vascular leakage caused by LPS, as evidenced by the Evans blue dye technique. Furthermore, histopathologic studies showed that IGF‐I decreased the proportion of hepatocytes with ballooning degeneration. Finally, IGF‐I also increased the expression of the hepatic growth factor (HGF) and the receptor for the epidermal growth factor (EGFR), markers of liver regeneration. Collectively, our data suggest that a combination of IGF‐I and BTP‐2 is a promising therapy for acute liver injury.

**Abbreviations**
AP1: activating protein‐1
BTP: 3,5‐bis(trifluoromethyl)pyrazole
CD‐14: cluster of differentiation 14
CD‐31: cluster of differentiation 31
cDNA: complementary DNA
COL‐1A: collagen type‐1 alpha
Cox‐2: cycloxygenase‐2
CRAC: calcium release‐activated channel
CREB: cAMP‐response element‐binding protein
EBD: evans blue dye
EGFR: epidermal growth factor
FDA: food and drug administration
GAPDH: glyceraldehyde 3‐phosphate dehydrogenase
H&E: hematoxylin and eosin
HGF: hepatic growth factor
IFN‐γ: interferon‐gamma
IGF‐I: insulin like growth factor‐I
IL‐1β: interleukin‐1 beta
IL‐6: interleukin 6
IL‐17: interleukin‐17
IP: intraperitoneal
IRF‐3: interferon regulatory factor 3
Kg: kilogram
LCN2: lipocalin‐2
LPS: lipopolysaccharide
MD 2: myeloid differentiation factor 2
Mg: milligram
Min: minute
mL: mililiter
m‐RNA: messenger ribonucleic acid
Nfat: nuclear factor of activated T‐cells
Nf‐KB: nuclear factor‐kappa B
NGAL: neutrophil gelatinase‐associated lipocalin‐2
Ng: nanogram
PBS: phosphate‐buffered saline
PECAM: platelet endothelial cell adhesion molecule‐1
q‐PCR: quantitative polymerase chain reaction
ROS: reactive oxygen species
RT‐qPCR: reverse transcriptase‐polymerase chain reaction
SERCA: sarcoendoplasmic reticulum (SR) calcium transport ATPase
SIRS: systemic inflammatory response syndrome
SOCE: store operating calcium entry complex
SOD: superoxide dismutase
TLR‐4: toll‐like receptor‐4
TNF‐α: tumor necrosis factor‐alpha
TRP: transient Receptor Potential
VEGF: vascular endothelial growth factor

## 1 INTRODUCTION

Acute liver injury can be caused by several conditions, including hepatitis, acetaminophen poisoning, and systemic inflammatory response syndrome (SIRS).^1^, ^2^, ^3^ Currently, there is no specific therapy for acute liver injury; however, the ability of the liver to regenerate is a unique asset to this organ. Nonetheless, liver inflammation caused by liver injury can lead to severe fibrosis and declining liver function, requiring liver transplantation.

Our goal is to develop a therapy to treat the acute liver injury in a mouse model induced by lipopolysaccharide (LPS). *Escherichia coli* and the LPS on its membrane are known to produce acute liver injury, especially in those with chronic liver disease.^4^ LPS initiates cellular toxicity by binding to and activating the TLR 4 complex, which downstream leads to an increase in cytosolic calcium that mediates many of the adverse effects of LPS on cellular functions,^5^, ^6^ such as enhanced inflammatory cytokine production.^7^ In response to LPS, the liver cells, such as Kupffer cells, produce pro‐inflammatory cytokines, including TNF‐α and IL‐1.^8^

To treat the acute liver injury induced by LPS in mice, we elected to utilize two therapeutic agents, IGF‐I and BTP‐2. IGF‐I therapy was selected because there was a reduction in serum IGF‐I levels following LPS injection in mice,^9^ which indicated liver injury because the liver is the primary source of circulating IGF‐I.^10^ In addition, we found that the decrease in serum IGF‐I in sepsis was associated with increased levels of circulating inflammatory cytokines.^11^ Collectively, our findings and others support the concept that the elevated pro‐inflammatory cytokine levels following LPS injection suppress the expression of growth hormone in the liver, decreasing serum IGF‐I.^11^, ^12^

The significance of the above findings can be perceived via the pleiotropic IGF‐I functions. With systemic LPS administration, there is usually a component of vascular injury.^13^, ^14^ IGF‐I has several functions that could counteract the effect of LPS to impair vascular function.^15^ IGF‐I increases the expression of endothelial adhesion molecules, the proliferation of resident endothelial progenitor cells, and the expression of the 25‐hydroxyvitamin D 1α‐hydroxylase that produces the vascular protective active metabolite of vitamin D. In addition, IGF‐I has many repair functions.^16^ For example, we recently showed that IGF‐I improved kidney and lung tissue repair.^9^, ^11^

BTP‐2 (YM‐50483) was selected for the therapy of LPS‐induced inflammation because it is a specific inhibitor of Orai 1 and 2. In this regard, Orai 1 and 2 form the core of the calcium release‐activated channel (CRAC) that promotes calcium influx through the plasma membrane.^17^, ^18^, ^19^ Furthermore, Orai 1 and 2 and the CRAC channel are functional components of store‐operated calcium entry (SOCE).^20^ Thus, BTP‐2 is a potent inhibitor of calcium influx^21^ and can block the calcium‐dependent, LPS‐induced detrimental effects on cell functions, including liver injury. Notably, BTP‐2 has been used successfully in experimental animals to suppress the immune system in severe inflammatory conditions, including acute liver injury.^22^

This study builds on our previous work in our mouse model of systemic LPS administration to evaluate kidney and lung responses.^9^, ^23^ This approach has a significant advantage in that it allows us to compare, in the same animals, the effects of our therapies on LPS‐induced toxicity on three major organs, the kidney, lung, and liver.

The main objectives of the current study were to determine the LPS toxicity on the liver and the therapeutic effects of IGF‐I and BTP‐2. For LPS‐induced toxicities, we sought to determine if (1) all the effects of LPS were a consequence of the various components of the TLR 4 receptor, (2) LPS increased all of the transcription factors known to be involved in inflammation, and (3) LPS had adverse effects on selected categories of liver mechanistic responses, including TLR 4 complex components, intracellular calcium signaling pathways, pro‐inflammatory expression, vascular integrity parameters, tissue damage, and tissue repair parameters. For the therapeutic effects of IGF‐I and BTP‐2, we evaluated their effects on LPS‐induced adverse changes. In addition, because IGF‐I has been shown to promote liver regeneration,^24^, ^25^ we also sought to determine if the liver‐restorative effect of IGF‐I acted through an increased expression of hepatic growth factor (HGF) and epidermal growth factor receptor (EGFR).^25^, ^26^

## 2 METHODS

### 2.1 Experimental design

**FIGURE 1:**
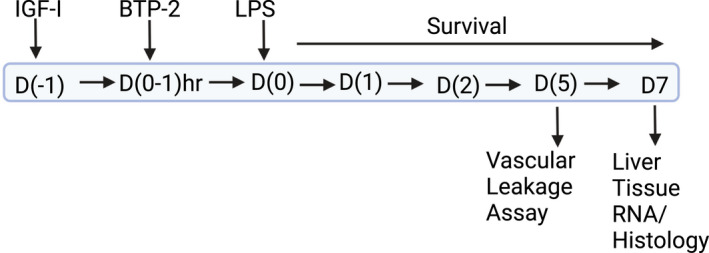
Schematic representation of the experimental setup used for the liver injury studies.

The experimental design is shown in Figure 1.

### 2.2 Animals

Female C57/BL6 mice were purchased from The Jackson Laboratory (Bar Harbor, ME, USA). All mice were used at ages 5–8 weeks. The investigators adhered to the Animal Welfare Act Regulations and other Federal statutes relating to animals and experiments involving animals and the principles outlined in the current version of the Guide for Care and Use of Laboratory Animals, National Research Council. All experiments were performed following the protocols approved by the Institutional Animal Care and Use Committee at Loma Linda University.

### 2.3 Acute liver injury induced by LPS

Female C57BL/6 mice received a sublethal or lethal dose of LPS from *Escherichia coli* (0127: B8 strain, Sigma Aldrich, St. Louis, MO, USA). LPS in sterile PBS was administered intraperitoneally at 20‐ or 25‐mg/kg body weight.

### 2.4 Treatment

Lenti‐IGF‐I was administered intramuscularly 24 h before LPS injection. In addition, the Trp and Orai calcium channel blocker BTP‐2 (YM‐58483 16 mg/kg, Cayman Chemical, Ann Arbor, MI, USA) was given IP 1 h before LPS injection.^9^, ^27^ Some of the animals received a combination of lenti‐IGF‐I and BTP‐2. Mice in control groups were injected with an equal volume of PBS.

### 2.5 Measurements of mRNA expression in the liver

To measure the mRNA expressions of the whole liver tissues, we performed RT‐qPCR. Liver tissues were snap‐frozen in liquid nitrogen. According to the manufacturer's instructions, total RNA was isolated using the RNeasy Micro Kit® (Qiagen, Valencia, CA, USA). First‐strand cDNA was synthesized using the SuperScript® III Reverse Transcriptase (Life Technologies, Grand Island, NY, USA). Quantitative RT‐qPCR was performed and analyzed in an Applied Biosystems 7900HT Real‐Time PCR machine (Applied Biosystems, Foster City, CA). The PCR condition was 10 min at 95°C followed by 40 cycles of 10 s at 95°C and 15 s at 60°C. The relative amount of mRNA was calculated using the comparative C_t_ (∆∆C_t_) method. All specific amplicons were normalized against GAPDH. Gene‐specific primers used in the current study are listed in Table S1.

### 2.6 Vascular leakage permeability assay

Evans blue dye (EBD) was dissolved in a 0.9% saline solution at a 5 mg/mL concentration and injected into the mouse tail vein (50 mg/kg, i.v). After 30 min, the liver tissues were harvested, dried, and weighed. Dried tissues were soaked in 3 ml of formamide and homogenized using a homogenizer followed by incubation at 60°C for 18 h. The homogenized tissues were centrifuged at 12 000 × *g* for 30 min. The absorbance of the supernatants was measured at 630 on a plate reader.^9^, ^28^

### 2.7 Liver histology

Serial tissue sections from 1 portion of the liver were stained with Masson trichrome stain and hematoxylin–eosin (H&E). The stained liver sections were imaged on an Olympus BX51 microscope, 40× magnification (Olympus, Center Valley, PA, USA). The captured images were further examined for liver morphology, hepatocyte ballooning, nuclear size changes, signs of necrosis, and apoptosis on ImageJ (National Institutes of Health, Bethesda, MD, USA). A minimum of 10 fields for each liver slide were examined for pathological injury quantification.

Masson trichrome‐stained sections were examined for collagen deposition in the liver tissues. The images were captured with a BIOREVO BZ7000 fluorescent microscope (Keyence).

### 2.8 Statistical analysis

Statistical analyses were performed with GraphPad software (Prism 5.02, San Diego, CA, USA). The quantitative analyses, such as qPCR data, were reported as the mean ± SEM and analyzed using one‐ or two‐way ANOVA, followed by a Dunnett's multiple comparisons test or a Bonferroni post‐hoc analysis unpaired *t*‐test. Evaluation of the histopathology preparations was blind, and specimen identity was revealed only after completion of the analyses. A *p*‐value of <.05 was considered to be statistically significant.

## 3 RESULTS

### 3.1 Previously published work on serum IGF‐I is applicable to our current liver injury study

The acute liver injury study utilized the liver samples obtained from the same animals in our previous studies of acute kidney injury and acute lung injury.^9^, ^23^ In these animals, we found that the serum IGF‐I level was significantly reduced after injection with LPS.^9^, ^11^, ^23^ To correct this deficiency, we engineered a lentiviral vector overexpressing IGF‐I. The lentiviral vector was then given to the mice intramuscularly at the beginning of the experiment to correct the IGF‐I deficiency. Under normal conditions, serum IGF‐I is bound to serum proteins, including IGF binding proteins, preventing IGF‐I, a small molecule, from being eliminated in the urine. However, pro‐inflammatory cytokines can suppress the expression of the growth hormone receptor in the liver, resulting in a decrease in the production of IGF‐I and its binding proteins. The reduction in IGF‐I binding proteins shortens the serum half‐life of IGF‐I.^11^, ^12^ Our gene therapy approach corrected the serum IGF‐I deficiency after IGF‐I gene therapy, serum IGF‐I increased to 400 ng/mL (a normal value) compared to 80 ng/mL before gene therapy.^9^, ^23^

### 3.2 Current studies on the liver

#### 3.2.1 LPS receptor

**FIGURE 2:**
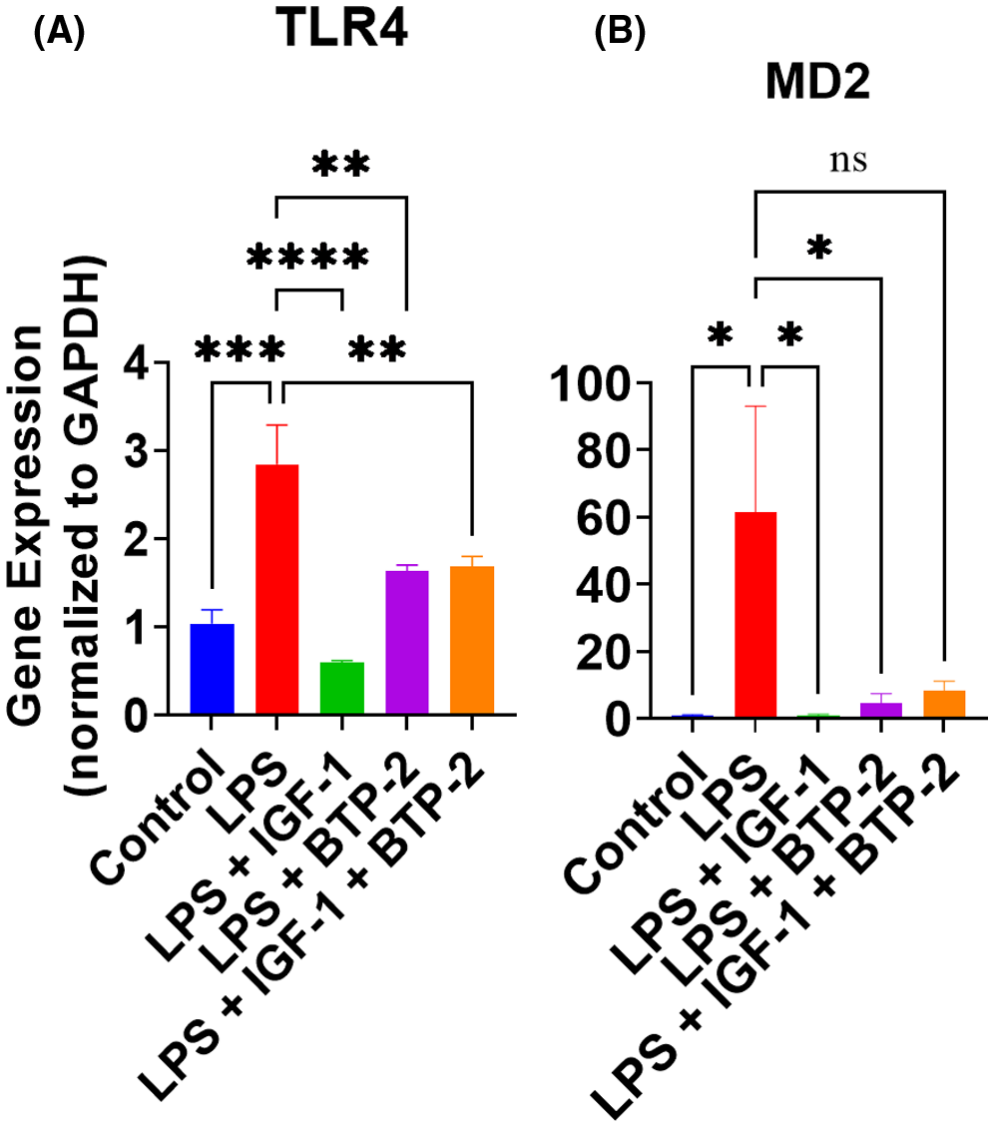
Effects of IGF‐I, BTP‐2 monotherapies, and IGF‐I + BTP‐2 combination therapies on the mRNA expression of the major regulator of TLR‐4 pathway. Day 7 RT‐qPCR of the markers (A) TLR‐4 and (B) MD2 are shown. Data are the mean ± SEM. *p < .05, **p < .01, ***p < .001, and ****p < .0001, ns, no significance. One‐way ANOVA, followed by Bonferroni's multiple comparisons test (*n* = 3).

LPS binds to the TLR 4 complex, which is composed of 2 molecules of TLR 4 that are dimerized by MD2. CD14 then binds to dimerized TLR 4 to form the complete TLR 4 complex. We found that LPS caused a significant increase in TLR 4 and MD 2. We did not measure CD14 gene expression because, in mice treated with LPS, the maximum increase in CD14 is seen in about 12 hours,^29^ whereas our measurements were only done at 7 days. Treatment of LPS animals with IGF‐I, BTP‐2, or the combination therapy significantly decreased TLR 4 expression (Figure 2A). MD 2 gene expression showed a similar profile for LPS and the treatment groups (Figure 2B). The mechanisms for the decreased expression of these two TLR 4 receptor components are not clear. However, the BTP‐2 finding provides evidence that the improvement in TLR 4 expression is, at least in part, a consequence of improvements in calcium signaling.

#### 3.2.2 Intracellular calcium signaling pathways

**FIGURE 3:**
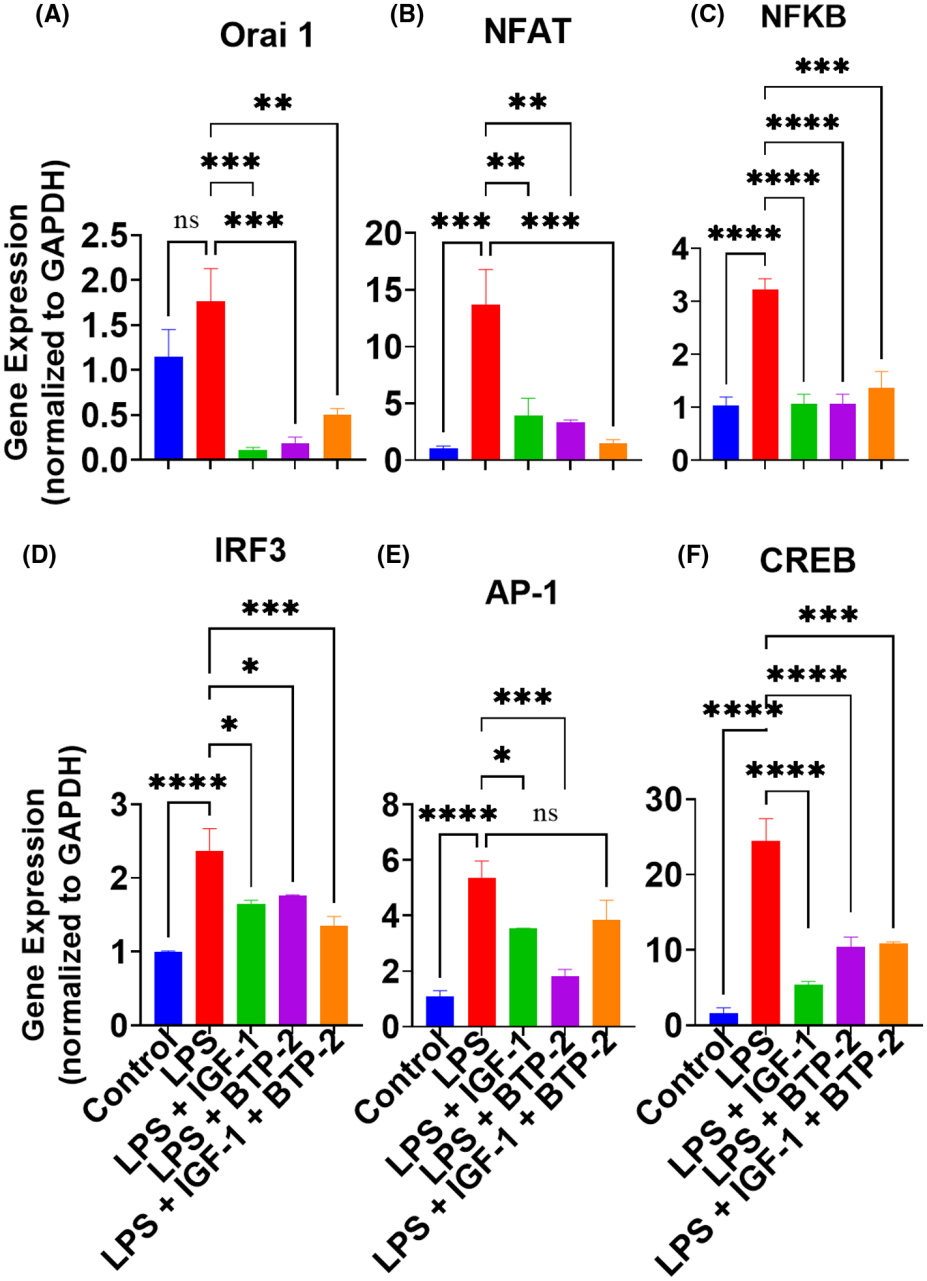
Effects of IGF‐I, BTP‐2 monotherapies, and IGF‐I + BTP‐2 combination therapies on the mRNA expression of calcium channel regulators in the livers. Day 7 RT‐qPCR of the markers (A) Orai 1, (B) Nfat‐1, (C) NFKB, (D) IRF‐3, (E) AP‐1, and (F) CREB. qPCR data are the mean ± SEM. *p < .05, **p < .01, ***p < .001, and ****p < .0001, ns, no significance. One‐way ANOVA, followed by Bonferroni's multiple comparisons test (*n* = 3).

TLR‐4 signaling increases cytosolic calcium through activating the release of ER calcium stores and the coordinated activation of the Orai 1/CRAC channels.^30^ The target of BTP‐2 is Orai 1 and 2. Both treatment groups with BTP‐2 show significant decreases in Orai 1 expressions at 7 days after initiating therapy (Figure 3A), suggesting that BTP‐2 has a relatively long half‐life and that there is a feed‐forward impact of Orai 1 and 2 on Orai 1 expression. Interestingly, IGF‐I also decreased Orai 1, which has not been reported. The mechanism for this action of IGF‐I is unknown. However, IGF‐I alone without BTP‐2 is sufficient to produce liver regeneration.

A pivotal transcription factor downstream from Orai 1 activation and SOCE is Nfat. All three treatment groups, i.e., IGF‐I alone, BTP‐2 alone, and the combination group, exhibited significantly decreased Nfat gene expression (Figure 3B). The above‐observed effects on Nfat are crucial because increases in Nfat lead to the expression of pro‐inflammatory cytokines. In general, changes in SOCE, as reflected by the gene expression of Orai 1, are attended by similar changes in Nfat I.^30^ However, the effect of IGF‐I to markedly decrease the effect of LPS to increase Nfat is also a novel finding. Because IGF‐I treatment was also associated with a decrease in Orai 1, it is likely that one of IGF‐I's actions is to decrease SOCE.

The results mentioned above suggest that LPS activates TLR 4, which leads to an increase in cytosolic calcium and the transcription factor, leading to the increased expression of most pro‐inflammatory cytokines, such as TNF‐α and IFN‐γ.^31^, ^32^ In addition to the Nfat, other transcription factors, for example, NFkB, IRF3, AP‐1, and CREB, are also associated with increased expression of pro‐inflammatory cytokines.^33^, ^34^ Consistent with the previous findings, we found that LPS increased the expression of liver NFκB which was markedly decreased to almost normal by all three treatment modalities (Figure 3C). In addition, LPS treatment increased the expression of IRF3 (Figure 3D), AP‐1 (Figure 3E), and CREB (Figure 3F), all of which were improved by the three treatment modalities (Figure 3D–F).

#### 3.2.3 Pro‐inflammatory cytokines

**FIGURE 4:**
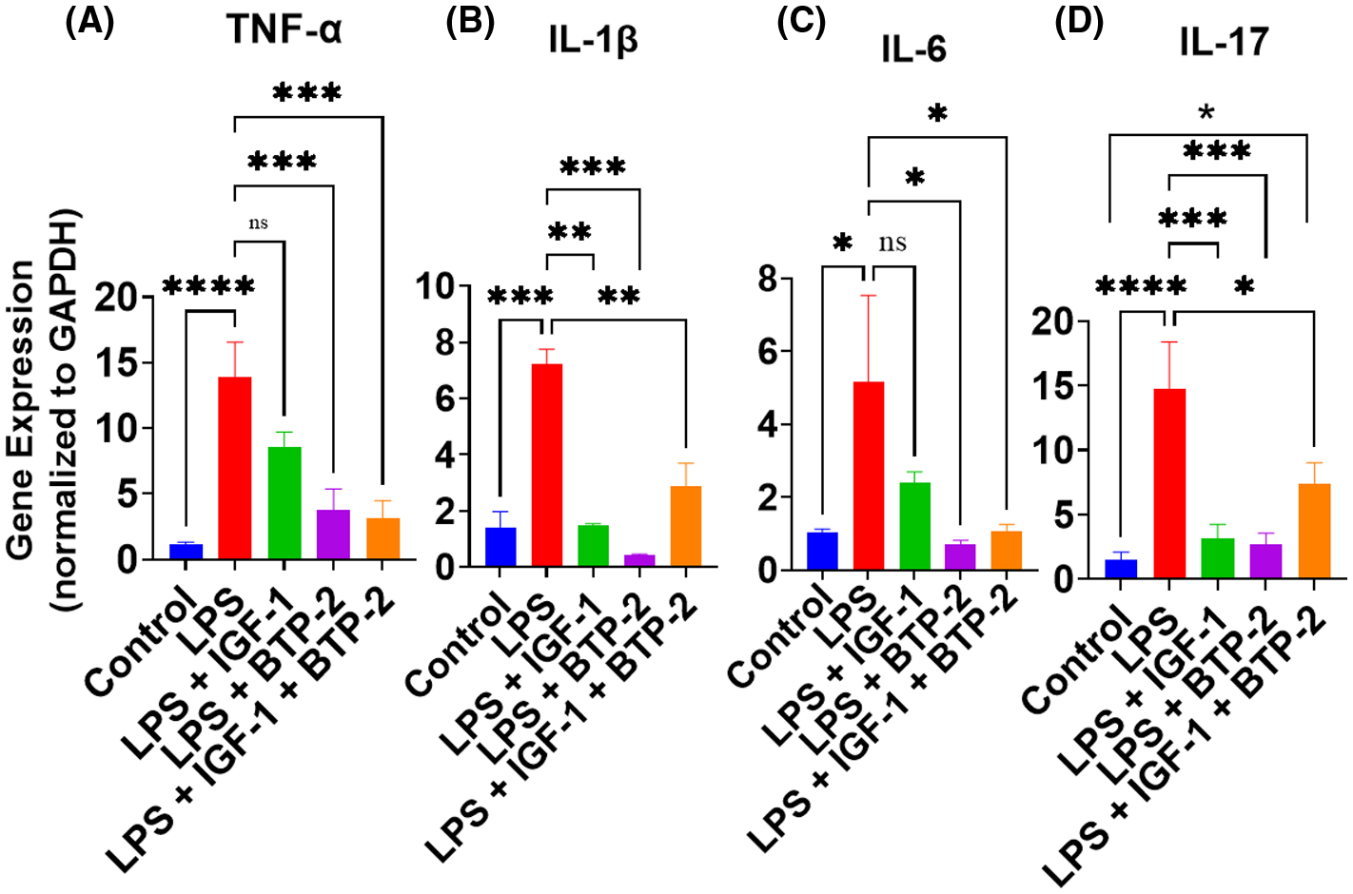
Effects of IGF‐I, BTP‐2 monotherapies, and IGF‐I + BTP‐2 combination therapies on the mRNA expression of major inflammatory markers. Day 7 RT‐qPCR of the markers (A) TNF‐α, (B) IL‐1β, (C) IL‐6, and (D) IL‐17. qPCR data are the mean ± SEM. *p < .05, **p < .01, ***p < .001, and ****p < .0001, ns, no significance. One‐way ANOVA, followed by Bonferroni's multiple comparisons test (*n* = 3).

LPS increased the gene expression of TNF‐α, which was significantly decreased by BTP‐2 and IGF‐I+ BTP‐2 but not by IGF‐I alone (Figure 4A). The lack of effect of IGF‐I alone could be due to an inadequate sample size. LPS increased IL‐1β, and this increment was decreased to normal in all three treatment groups (Figure 4B). IL‐6 gene expression was decreased to normal in the two groups containing BTP‐2 but not that with IGF‐I alone (Figure 4C). IL‐17 has been associated in the past with post‐inflammatory fibrosis.^32^ Our study showed a marked increase in IL‐17 gene expression in response to LPS, which was attenuated by all three treatment groups (Figure 4D). Cytosolic calcium changes are thought to be pivotal in determining pro‐inflammatory cytokine production.^7^ In this regard, both IGF‐I and BTP‐2 decrease the effect of LPS to increase Orai. However, in general, there were differences in the degree of magnitude of suppression of pro‐inflammatory cytokines between IGF‐I and BTP‐treated groups. This may be partly because IGF‐I also has additional effects on calcium signaling.^35^ These data support the combinatorial therapy because IGF‐I may be less effective in suppressing the expression of pro‐inflammatory cytokines.

#### 3.2.4 Vascular integrity

**FIGURE 5:**
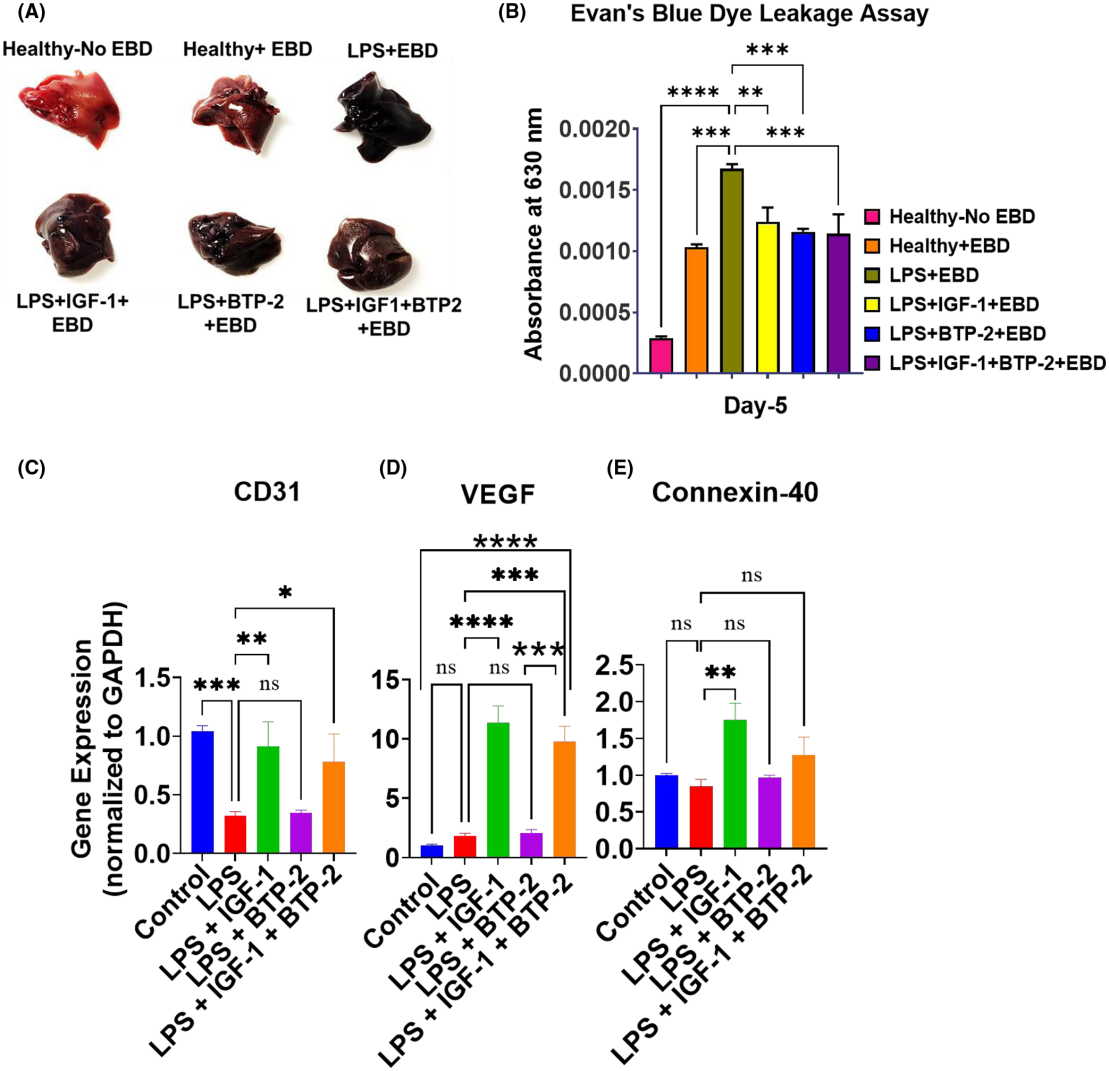
Effects of the IGF‐I and BTP‐2 monotherapies and the IGF‐I + BTP‐2 combination therapy on LPS‐induced acute liver injury‐associated vascular integrity. (A). Evans blue dye‐stained Liver tissues. (B) Evans blue dye leakage measurements of vascular leakage in the Liver. Day 7 RT‐qPCR for the vascular markers (C) CD31, (D) VEGF, and (E) Connexin‐40 (Data are the mean ± SEM. *p < .05, **p < .01, ***p < .001, ****p < .0001 and ns, no significance. One‐way ANOVA, followed by Dunnett's multiple comparisons test (*n* = 3).

Vascular integrity was interrogated by performing functional analysis using an Evans blue dye (EBD) retention assay. On day 5 post‐LPS and therapy, there was a significant increase in EBD retention in the LPS group, while all three treatment groups decreased EBD retention (Figure 5A,B).

We found CD31 (PECAM‐1) gene expression was markedly decreased by LPS, a change which was improved by treatment with IGF‐I as well as the combination of IGF‐I and BTP‐2 but not by BTP‐2 monotherapy (Figure 5C). These data also support the combinatorial therapy. IGF‐I but not BTP‐2 can protect the vascular integrity and promote liver regeneration. On the other hand, VEGF gene expression was increased by LPS, but the change was not significant (Figure 5D). The gene expression of Connexin‐40, a gap junction protein essential for endothelial cell function, was unchanged by LPS treatment (Figure 5E), whereas previous workers found that vascular damage caused by LPS was connexin‐40 dependent.^36^, ^37^

#### 3.2.5 Tissue damage/repair

**FIGURE 6:**
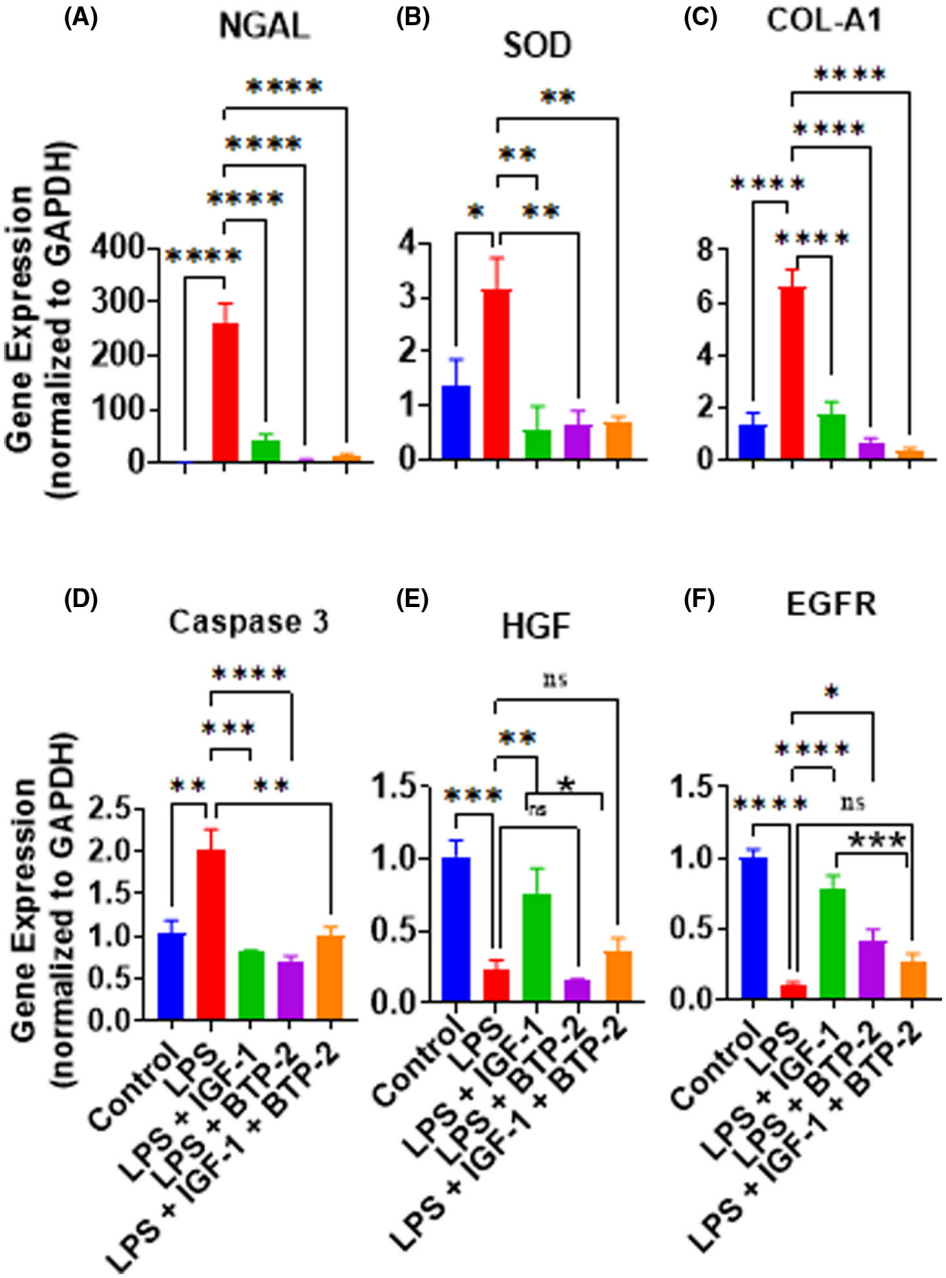
Effects of the IGF‐I and BTP‐2 monotherapies and the IGF‐I + BTP‐2 combination therapy on LPS of the injury and repair markers in the liver. Day 7 RT‐qPCR for the markers (A) NGAL, (B)SOD, (C) Collagen‐1, (D) Caspase 3, and liver regeneration marker (E) HGF and (F) EGFR. Data are the mean ± SEM. *p < .05, **p < .01, ***p < .001, ****p < .0001 and ns, no significance. One‐way ANOVA, followed by Bonferroni's multiple comparisons tests (*n* = 3).

NGAL (lipocalin‐2) is associated with kidney and cardiac tissue injury.^38^ In our study, LPS treatment leads to an enormous increase in the expression of NGAL in the liver (Figure 6A). Treatment with IGF‐I and the combination of IGF‐I and BTP‐2 markedly decreased NGAL gene expression. We next evaluated the effects of LPS and treatment modalities on reactive oxygen species (ROS). In this regard, we found that superoxide dismutase (SOD) gene expression was significantly increased in the LPS group and was returned to normal in all three treatment groups at day 7 (Figure 6B). There was a marked increase in LPS treatment of type I collagen gene expression, which could be an early marker of post‐inflammatory fibrosis. Type I collagen expression was reduced to baseline levels by all three treatments (Figure 6C). In addition, LPS treatment increased caspase 3 gene expression significantly, which was decreased to normal in all treatment groups (Figure 6D). Caspase 3 is known to be associated with apoptosis.

The liver is unique among the organs as it can regenerate itself.^39^ Therefore, we sought to evaluate the effects of LPS and the treatment on liver regeneration. The prominent markers for liver regeneration include the HGF and EGFR.^25^, ^26^ Instead of inducing regeneration, LPS markedly decreased the expression of these regeneration genes (Figure 6E,F), suggesting an impairment in self‐repair. Interestingly, treatment with IGF‐I substantially increased the mice substantially increased the HGF and EGFR gene expression (Figure 6E,F). However, treatment with BTP‐2 alone or in combination with IGF‐I negatively influenced the beneficial effects on IGF‐I toward the liver regeneration marker.

#### 3.2.6 Histopathology

**FIGURE 7:**
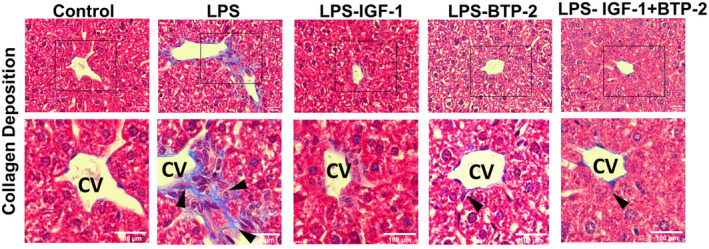
Effects of the IGF‐I and BTP‐2 monotherapies and the IGF‐I + BTP‐2 combination therapy on LPS‐induced acute liver injury‐associated fibrosis. Images of trichrome‐stained liver sections show a characteristic pattern of fibrosis. There is scarring (arrowheads), around the central vein (CV), and fibrosis is indicated by the presence of blue collagen fibers stained strongly with Trichrome stain, specifically in LPS group (scale bar 100 μm).

Masson trichrome‐stained liver sections indicated a presence of blue color‐stained collagen fibers against a red background of hepatocytes highlighting the presence and distribution of reactive fibrosis resulting from liver injury.^40^ Images of trichrome‐stained liver tissue in LPS groups specifically exhibited a characteristic pattern of fibrosis (Figure 7).

**FIGURE 8:**
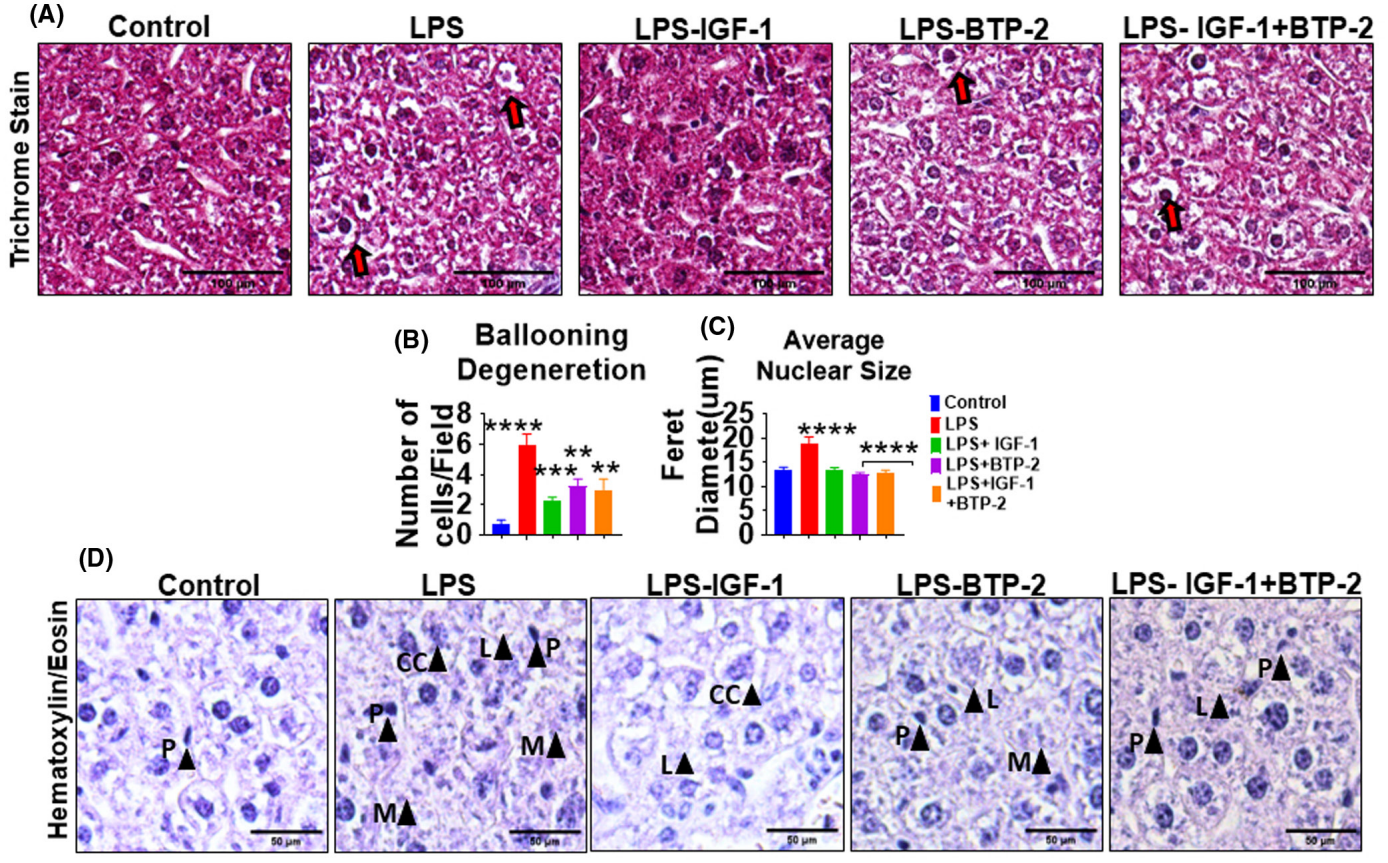
Effects of the IGF‐I and BTP‐2 monotherapies and the IGF‐I + BTP‐2 combination therapy on LPS‐induced acute liver injury‐associated cell injury. A) Trichrome stain images of liver section show ballooning cell degeneration (red arrows), (B) Quantification of ballooning degeneration of cells as indicated by the number of damaged cells per field (C) increased nuclear size indicating cellular damage and injury, especially in the LPS group. (Scale bar‐100 um) and (D) Hematoxylin–eosin staining images of liver sections exhibiting signs of apoptosis such as chromatin condensation (CC), condensation of the nucleus/pyknosis (P), and signs of necrosis such as loss of plasma membrane integrity (M), and loss of cellular organelles (L) specifically in LPS groups (scale bar 50 μm).

The main histopathologic abnormality in the LPS‐treated group was increased hepatocyte ballooning, a unique type of cell death occurring primarily in the liver.^41^ When dehydrated during histological preparation, the ballooning cells show specific morphological features that include clear cytoplasm or empty cell sacs with a central nucleus. The LPS‐treated liver samples exhibited a marked increase in ballooning cells compared to the control and treatment groups (Figure 8A,B). Another histopathologic feature of the damage by LPS was a significant increase in nuclear size (Figure 8C). The relationship between nuclear size and cell damage has been studied in vitro in cells induced to undergo necrosis.^42^ The increase in cell nuclear size serves as a marker of tissue damage‐induced inflammation.^43^

Integrating our histopathologic findings with molecular findings, we find evidence of apoptosis such as chromatin condensation, condensation of the nucleus (pyknosis), and loss of water resulting in significant cell shrinkage.^44^, ^45^ We also observed signs of necrosis as evident by loss of plasma membrane integrity^46^ and disruption of cell organelles^47^ specifically in LPS groups (Figure 8D). This was attended by an increase in molecular evidence of fibrosis as evidenced by an increased gene expression and histological staining for type I collagen (Figures 6C and 7). Necrosis is known to produce inflammation.^48^ However, we found minimal histopathologic evidence of inflammation despite the apparent evidence of tissue damage and expression of pro‐inflammatory markers (Figure 4).

## 4 DISCUSSION

In the past work, we studied the effects of individual BTP‐2 or IGF‐I and combination therapy on acute kidney injury and acute lung injury induced by LPS in mice in vivo.^9^, ^23^ In these past studies, we measured the effect of these therapies on TLR 4 expression (receptor for LPS), calcium signaling, pro‐inflammatory cytokine production, and tissue damage/repair in the isolated kidneys and lungs. In all categorical measurements, there was an improvement with either in vivo treatment with IGF‐I, or BTP‐2 alone, or their combination.^9^, ^23^

In the present study of acute liver injury, the same therapeutic response parameters as in our earlier studies were evaluated, including Evans blue dye (EBD) retention, histopathology for hepatic cell ballooning, and regenerative gene responses.

### 4.1 TLR 4 signaling

Our objective was to determine the effects of LPS on all components of the TLR 4 complex and the effect of our therapy on these components. Several reports suggest TLR 4 knockout mice have attenuated inflammation.^6^, ^49^ The TLR 4 signaling complex includes CD14 and MD2.^50^ We found that TLR 4 and MD2 were both markedly increased by LPS. However, there were significant decreases in these two signaling agents in all three therapeutic groups. MD2 dimerizes two molecules of TLR 4 in preparation for endosome internalization and activation of downstream pathway elements. CD14 acts as a co‐receptor for TLR 4 and MD2 to detect bacterial LPS. CD14 binds to TLR 4 and facilitates the endocytosis of TLR 4.^51^ We did not measure liver CD14 gene expression because it has a maximum effect at about 12 hours,^29^ whereas our measurements were all made at day 7. With respect to the effect of our therapy on CD14, CD14 is important for the TLR 4 endocytosis, and we found that TLR 4 was decreased by our therapies, suggesting that our therapies were effective on the endocytosis action of CD14. However, there is also a soluble form of CD14, which may have independent actions of the TLR 4 complex.^50^ Our study did not address these soluble actions of CD14, and therefore it is possible that soluble CD14 has an inflammatory action not addressed by our therapies.

### 4.2 Calcium signaling

TLR 4 activation leads to an increase in cytosolic calcium.^52^ One of the major determinants of cytosolic calcium is SOCE, of which Orai 1 and 2 are regulatory components. LPS significantly increased the gene expression of Orai 1, which was markedly decreased to below normal in all three treatment groups. This was expected in the BTP‐2 treatment groups because BTP‐2 is a specific inhibitor of Orai 1 and 2. The effect of LPS to increase the gene expression of Orai 1/SOCE leads to an increase in cytosolic calcium.^53^, ^54^, ^55^, ^56^, ^57^, ^58^ The increase in cytosolic calcium caused by LPS led to an increase in Nfat gene expression, a calcium‐dependent transcription factor.^59^ In the liver, LPS caused a marked increase in Nfat, which was corrected in all three treatment groups after 7 days of therapy. The effect of IGF‐I therapy to decrease Orai 1 was an unexpected and novel finding. However, the effects of IGF‐I on cytosolic calcium are complex because IGF‐I also affects SERCA, which tends to increase cytosolic calcium.^35^ Our data and others provide strong evidence for the conclusion that the increase in pro‐inflammatory cytokines in response to stresses such as LPS is mainly due to their ability to increase cytosolic calcium.^9^, ^23^, ^55^, ^60^, ^61^

### 4.3 Transcription factors related to the production of pro‐inflammatory cytokines

Increased cytosolic calcium leads to an increase in Nfat, which increases several calcium‐dependent transcription factors that regulate pathways, including pro‐inflammatory cytokine production.^7^, ^30^ These transcription factors include NFkB, CREB, and AP‐1, which in turn stimulate the inflammasome, interleukins, TNF‐α, Cox 2, and IRF3, that act to regulate IFN‐γ.^62^ We found that all of these transcription factors activated by LPS were significantly reduced by our three therapy groups, and in particular, BTP‐2 therapy. Therefore, it seems likely that the decrease in these transcripts by our therapy is responsible for the decreases in inflammatory cytokines described in the current study.

Thus far, we have confirmed that the TLR 4 complex largely initiates acute liver injury. The downstream effects of TLR 4 include actions on calcium signaling and calcium‐dependent transcription factors, which are remarkably pleiotropic. In this regard, it seems likely that many of the favorable changes in LPS‐induced damage are a consequence of the action of BTP‐2 to inhibit SOCE.

### 4.4 Pro‐inflammatory cytokine production

Our therapy counteracts the effect of LPS on the TLR 4 complex, the multiple transcription factors mentioned above, and several pro‐inflammatory cytokines.

Accordingly, the following pro‐inflammatory cytokines were increased by LPS and decreased by our three therapeutic modalities: TNF‐α, IL‐1β, IL 17, and IL‐6, a finding confirming the work by others.^33^, ^63^, ^64^, ^65^ Cytosolic calcium reduction, specifically by BTP‐2, accounts for all of the inhibitions of these pro‐inflammatory cytokines. Furthermore, the transcription factors NFκB, AP‐1, CREB, and IRF3 have several other pro‐inflammatory actions in addition to the upregulation of pro‐inflammatory cytokines.^65^, ^66^, ^67^ Interestingly, Nfat and AP‐1 interact to produce appropriate immune responses.^68^ Accordingly, it is possible that the effect of our therapies to reduce these LPS‐stimulated transcription factors, apart from their effect on pro‐inflammatory cytokines, could contribute to the favorable effect of our therapies on vascular integrity and tissue damage, as described below.

### 4.5 Vascular integrity

In our study of acute kidney injury, the Evans blue dye exclusion test showed substantial renal leakage in mice treated solely with LPS^9^ and significant improvement in vascular leakage by all three treatment modalities. PCR measurements indicated a decrease in CD31 in response to LPS, significantly improved by IGF‐I treatment. LPS did not significantly increase VEGF gene expression but was markedly increased by IGF‐I therapy, possibly representing a compensatory response to vascular injury. IGF‐I also improved vascular leakage. Others have shown that LPS negatively impacts vascular integrity by decreasing connexin‐40 and resultant endothelial coupling^69^; however, we could not confirm this observation. Our major findings on vascular integrity were that IGF‐I treatment had a positive effect on Evans blue dye leakage and the expression of CD31/PECAM‐1 and possibly VEGF. Moreover, although BTP‐2 therapy reduced vascular leakage, the effects were not consistent with gene expression. These mixed results of vascular leakage and gene expression with our therapy emphasize the importance of future‐focused studies to disclose the mechanism of LPS‐induced impairment and vascular integrity and the specific roles of Orai 1 in those processes.

### 4.6 Liver damage

There are several risk factors for LPS‐induced acute liver injury. Pro‐inflammatory cytokines can cause liver injury^70^, ^71^ and decrease the production of IGF‐I by the liver.^72^ IGF‐I is essential for the normal conversion of 1,25‐dihydroxyvitamin D, the active metabolite of vitamin D, and an important regulator of cell differentiation.^43^ In our liver study, the profibrotic marker NGAL^38^ was markedly increased by LPS; moreover, all three treatment modalities reduced NGAL expression to normal or near‐normal. LPS also increased Superoxide dismutase gene expression; a change returned to control levels in all three of the treatment groups. Consistent with this profibrotic function for NGAL, we found that LPS caused a marked increase in collagen 1 (a marker for liver fibrosis), whereas all three of our treatments reduced this LPS‐induced increment to normal. Additionally, caspase 3 gene expression (involved in apoptosis) was significantly increased by LPS and attenuated in all three treatment groups. In aggregate, these molecular findings indicate that LPS causes substantial liver injury and suggests significant improvement in all three therapeutic groups as evaluated by the aforementioned markers of liver injury.

Integrating our histopathologic findings with molecular findings, we find evidence of necrosis and apoptosis, which were attended by an increase in molecular evidence of fibrosis (increased gene expression of type I collagen). The increase in cell nuclear size serves as a mechanotransducer of tissue damage‐induced inflammation.^43^ Necrosis is known to produce inflammation, however, we found minimal histopathologic evidence of inflammation despite the apparent evidence of damage.^48^ The inflammatory phase may have occurred early on, and by the time we made our measurements at 7 days, the inflammation may have subsided. This interpretation is consistent with a finding that major pro‐inflammatory cytokine gene expression levels were significantly reduced by 7 days in all three treatment groups. However, in the LPS‐untreated group at 7 days, proinflammatory cytokine was substantially elevated despite the fact that minimal immune cell infiltration was observed. The cell source for the high gene expression of proinflammatory cytokines will require future experimentation.

### 4.7 Liver repair

Because of its unique capacity for regeneration, we evaluated the effects of LPS toxicity on liver repair, and we anticipated that damage from LPS might induce some regeneration in the liver. However, two specific markers of liver regeneration, the gene expression of hepatic growth factor and EGF receptor, were decreased instead of increased by LPS treatment. Because LPS caused liver damage, we anticipated that both hepatic growth factor and EGF receptor gene expression would be increased, given the regenerative capacity of the liver.^73^ IGF‐I treatment did, however, effectively increase the expression of both hepatic growth factor and EGF receptor. That IGF‐I promotes liver regeneration has been established in the past. Our findings address one of the potential mechanisms whereby IGF‐I helps to restore liver function following damage.

LPS caused substantial liver damage, as indicated by the findings that gene expression of NGAL, caspase 3, SOD, and COL‐1A were markedly increased. Importantly, all of these adverse changes were normalized by the three therapies. Although IGF‐I was the only therapy that increased HGF and the receptor for EGF, IGF‐I was no more effective than the BTP‐2 therapeutic groups in correcting the increments and gene expression parameters representing tissue injury. Therefore, the discrete effects of IGF‐I to improve genetic markers of liver regeneration appear to be distinct from those mechanisms that induce liver damage that are influenced by therapies that include Orai 1/SOCE inhibition. In particular, IGF‐I was more effective than the BTP‐2 therapy groups in preventing the ballooning of hepatic cells, which is an adverse apoptotic action of LPS on the liver.^41^ Taken together, the genetic data and histologic data indicate that both IGF‐I and BTP‐2 therapies have a favorable effect on restoring liver function after LPS‐induced acute liver injury.

It will be important in future studies to evaluate the significance of the effects of IGF‐I therapy to promote HGF and EGFR, particularly since this therapy is an FDA‐approved medication. Moreover, liver injury in systemic inflammatory diseases increases the risk of sepsis,^74^ emphasizing the importance of effective liver therapy in patients with systemic inflammatory disease.

## AUTHOR CONTRIBUTIONS

Samiksha Wasnik, Xiaolei Tang, and David J. Baylink designed the research, Antoine Nehme, Mahdis Ghahramanpouri, Iqbal Ahmed, Mohadese Golsorkhi, Natasha Thomas, and Kevin Munoz performed research and acquired data, Samiksha Wasnik, Amir Abdipour, Sean M. Wilson, David J. Baylink analyzed the data and interpreted the results, David J. Baylink wrote the manuscript; and Samiksha Wasnik, Xiaolei Tang, and Sean M. Wilson performed the critical reading and editing of the manuscript.

## FUNDING INFORMATION

This research was supported by funds from the Department of Medicine at Loma Linda University.

## DISCLOSURES

No conflict of interest.

## Supporting information


Appendix S1

## Data Availability

The data that support the findings of this study are available in the methods and/or Supporting Information of this article.
