# Supplementary material for: Combination therapy of insulin‐like growth factor I and BTP‐2 markedly improves lipopolysaccharide‐induced liver injury in mice
Source: FASEB J. 2022 Jul 15;36(8):e22444. doi: 10.1096/fj.202200227RR (PMC12166284; doi:10.1096/fj.202200227RR)
Supplement: Supplementary file 1 — Appendix S1 [file FSB2-36-e22444-s001.docx]

**Supplementary Table**

| **Table S1 Primer List** | | |
| --- | --- | --- |
| **Gene** | **Forward** | **Reverse** |
| TLR-4 | GCCTTTCAGGGAATTAAGCTCC | GATCAACCGATGGACGTGTAAA |
| MD2 | AAGACTGAGGGGAACCAATG | CCATGGCACAGAACTTCCTT |
| ORAI 1 | CTCAACTCGGTCAAAGAGTCAC | CACGACCTCTGCTAGGAAAAG |
| NFATC1 | GGAGAGTCCGAGAATCGAGAT | TTGCAGCTAGGAAGTACGTCT |
| NFKB | ATGGCAGACGATGATCCCTAC | TGTTGACAGTGGTATTTCTGGTG |
| IRF3 | AACCGGAAAGAAGTGTTGCG | CCCTGGAGTCACAAACTCATAC |
| AP-1 | ACTCGGACCTTCTCACGTC | GGTCGGTGTAGTGGTGATGT |
| CREB | AGCAGCTCATGCAACATCATC | AGTCCTTACAGGAAGACTGAACT |
| TNF-A | CCTGTAGCCCACGTCGTAG | GGGAGTAGACAAGGTACAACCC |
| IL-1B | GAAATGCCACCTTTTGACAGTG | TGGATGCTCTCATCAGGACAG |
| IL-6 | CTGCAAGAGACTTCCATCCAG | AGTGGTATAGACAGGTCTGTTGG |
| IL-17 | GGCCCTCAGACTACCTCAAC | TCTCGACCCTGAAAGTGAAGG |
| CD-31 | CTGCCAGTCCGAAAATGGAAC | CTTCATCCACCGGGGCTATC |
| VEGF | GCACATAGAGAGAATGAGCTTCC | CTCCGCTCTGAACAAGGCT |
| CONNEXIN 40 | CCACATTCGTTATTGGGTACTGC | TACTGGGTACTCATAGGCACC |
| NGAL | GGGAAATATGCACAGGTATCCTC | CATGGCGAACTGGTTGTAGTC |
| SOD | AACCAGTTGTGTTGTCAGGAC | CCACCATGTTTCTTAGAGTGAGG |
| COL-I | TAAGGGTCCCCAATGGTGAGA | GGGTCCCTCGACTCCTACAT |
| CASPASE 3 | TGGTGATGAAGGGGTCATTTATG | TTCGGCTTTCCAGTCAGACTC |
| HGF | ACTTCTGCCGGTCCTGTTG | CCCCTGTTCCTGATACACCT |
| EGFR | GCCATCTGGGCCAAAGATACC | GTCTTCGCATGAATAGGCCAAT |
| GAPDH | TGGCCTTCCGTGTTCCTAC | GAGTTGCTGTTGAAGTCGCA |

Notes: TLR-4: Toll-like receptor 4, MD2: Myeloid differentiation factor-2, ORAI-1: ORAI calcium release-activated calcium modulator 1, NFATC1: Nuclear factor of activated T cells 1; NFKB: Nuclear factor kappa B, IRF3: Interferon Regulatory Factor 3, AP-1: Activator protein 1, CREB: cAMP Response Element-Binding Protein, TNF-α :Tumor Necrosis Factor -α, IL-1B: Interleukin 1 beta, IL-6: Interleukin-6; IL-17:Interleukin 17; CD-31: Platelet/endothelial cell adhesion molecule 1, VEGF: Vascular endothelial growth factor, NGAL: Lipocalin 2; SOD: Super Oxide Dismutase, Col1: Collagen type I, HGF: Hepatic growth factor, EGFR: Epidermal growth factor receptor, GAPDH: Glyceraldehyde-3-Phosphate Dehydrogenase
